# The family as provider of intergenerational support during COVID-19: a study into the mental health consequences for 65+ Europeans

**DOI:** 10.3389/fpubh.2024.1418472

**Published:** 2024-10-21

**Authors:** Lore Van Herreweghe, Wim Van Lancker

**Affiliations:** Centre for Sociological Research, University of Leuven, Leuven, Belgium

**Keywords:** intergenerational support, intergenerational relations, informal care, mental health, COVID-19

## Abstract

**Introduction:**

Intergenerational support is an important determinant of mental health. Due to limited access to formal care, the role of the family as provider of support became more prominent during the COVID-19 pandemic. To date, it remains unclear how intergenerational support from adult children to older parents was affected during the pandemic and whether this had consequences for the mental health of the parent generation.

**Methods:**

Using data from the Survey of Health, Ageing and Retirement in Europe (SHARE) Corona Surveys, we explore whether changes in support going from non-coresident adult children to their parents are associated with parents’ increase in depressive feelings. Additionally, we test whether the pandemic context and public health measures affected this relationship.

**Results:**

During the pandemic, families are found to provide more support. These changes in intergenerational support, however, were related to increased depressive feelings for the older parents. Furthermore, both the strictness of public health measures and the concurrent epidemiological situation affected this relationship.

**Conclusion:**

We conclude that the family is an essential source of late-life well-being, but stressful life events, such as public health crises, put pressure on these intergenerational relations with potential adverse mental health outcomes. Future policies should take into account the ambivalent nature of intergenerational relationships.

## 1 Introduction

2020 will forever be associated with the coronavirus pandemic. Physical distancing, stay-at-home orders and mandatory face masks were some of the policy responses implemented to slow down the spread of the coronavirus. After the first wave of infections in 2020, it became clear that more waves were likely to follow and that people would continue to live in such “new normal” in 2021 as well.

Due to their high risk profile, in about all countries, older adults were encouraged to self-isolate and to limit their contacts to the bare minimum (1). During this period, there was a strong focus on the often dramatic situations in nursing homes (2), but older adults living at home mainly flew under the radar. This despite the fact that epidemiological control measures restricted individuals’ access to physical forms of social interaction, while at the same time increased the demand for help and support (3). Given the strong connection between social interactions, social support and mental well-being, concerns arose that strict social distancing measures may put older adults at greater risk of depressive feelings, anxiousness and loneliness (4, 5). Therefore, this study centres on the relationship between changes in intergenerational support during the pandemic and the mental well-being of older adults.

This particular focus on intergenerational support exchanges is relevant since older adults living at home generally receive some sort of practical support (e.g., help in the household), often related to mobility limitations and declining health (6). A key source of such support is the family (7) and is considered to be a main determinant of late-life well-being (8, 9). However, intergenerational physical contacts were supposed to be drastically reduced as a direct consequence of the public health measures (10) or avoided out of fear of contamination (11).

Nevertheless, some studies found that, going against official guidelines, intergenerational contacts still remained prevalent, in some cases even more than in pre-pandemic times (3, 12–14). This may not only be important in order to fulfill the (increased) need for help and support, but also for buffering the potentially negative impact of the COVID-19 restrictions on the mental well-being of the older population (15). In contrast, based upon pre-pandemic studies, increased dependency on family members could also aggravate low mental well-being among the older population (16). Whether the negative effect of increased dependencies on adult children also pertains during a public health crisis such as the COVID-19 pandemic remains unclear. We address this research gap by questioning whether and how changes in receiving intergenerational support during the pandemic are related to increases in feelings of sadness and depression among the older European population.

## 2 Background

While governments implemented measures in order to contain the spread of COVID-19 and protect public health, results from studies conducted during the pandemic’s early stages signaled some negative psychosocial side-effects of the stay-at-home orders (17, 18). The pandemic, for example, posed some serious challenges to maintain intergenerational connections (11). This is concerning since these are crucial for mental health and well-being (8, 9), as well as important sources of support (7).

Caring for grandchildren, for example, was strongly discouraged, even though the need was high, due to the closure of schools and childcare services in many countries (19). Adhering to such guidelines by stopping or highly reducing the time spent looking after grandchildren, however, has negative consequences for the mental health of the grandparents, as pointed out by the study of Di Gessa et al. (20). The fact that grandparents were not able to see and take care of their grandchildren for reasons beyond their control was hypothesized to lead to increased levels of distress (20). Such mechanisms might also be relevant for intergenerational support going in the other direction, i.e., from adult children to their parents, but have never been tested in an European context.

Looking into support exchanges from adult child to parents during the pandemic, we would expect a general decline in these kind of interactions, due to the fear of infecting an older parent or getting infected by contact with a relative (11). This could be detrimental for those who rely on support and care provided by their children if the remaining amount does not meet their needs anymore (13). However, multiple studies proposed that parents and non-coresident children were actually more in touch with one another during the pandemic than before. This was possible due to a switch to alternative modes of communication (e.g., telephone and video calls) (10, 15), increasing concerns over each other’s health and well-being (21), and intensified exchanges of instrumental support (3, 12–14).

The latter is especially relevant as older parents are likely to rely more on their adult children due to COVID-19 restrictions (22). Activities such as grocery shopping, accessing healthcare and receiving home care posed risks, and became tasks that were preferably done by others (23). The reduced availability of paid services and care support due to COVID-19 related control measures (24), thus seemed to be compensated by increased levels of family care. It remains unclear, however, whether and how such compensations may impact the well-being of older adults.

Based upon pre-pandemic studies, we know that receiving support from adult children is predictive of better well-being for older adults (25) and especially for women (26). An important nuance, however, is that it might also result into increased feelings of dependence under certain conditions (8, 25). Reciprocity is considered one such important condition for promoting the well-being of both donor and receiver (27). Receiving support but not being able to give (equally) in return may translate into feelings of guilt and dependency and lower levels of life satisfaction (28). Fulfilling this reciprocity principle may become more difficult as the pandemic limits the short-term opportunities for older adults to support their family members in return (27). Additionally, receiving help from adult children reduces the parents’ sense of independence and autonomy, as it conflicts the norms associated with their parental role (16). In the context of COVID-19 and restricted availability of formal care services, feelings of dependency are likely to increase and might exacerbate the negative association between receiving instrumental support from kin and late-life well-being.

Moreover, the pandemic could also lead to rising conflicts and concerns within families (29). When adult children and parents are not agreeing on the attitudes toward COVID-19 and compliance toward preventive measures (30), this could translate into diverging expectations about support exchanges and negative interactions as well. For example, parents can expect their children to fill in the void in formal care while adult children want to adhere to the precautions and limit personal contact as much as possible. Such conflicts and disagreements may be more likely to occur when stringency levels are high and more containment rules are implemented, or be more intense when mortality rates are peaking. Pre-pandemic studies already illustrated the negative effect of conflict in intergenerational exchanges for older adults’ mental health (8), while more recent studies warned for the declining quality of intergenerational contacts during the pandemic (31). The ambivalent character of intergenerational relationships (32) might be exacerbated during a public health crisis. These moderating effects of the pandemic are already signaled by Jiang and Fung (27) for China, but are not yet considered in an European context.

In light of this, it is important to examine how family support networks have been affected by the pandemic and to what degree this has implications for the mental health of the care recipients, i.e., the older parents. In this study we explore whether and to what extent changes in receiving intergenerational support provided by adult children were associated with a decrease in the parent’s mental well-being during the pandemic. To our knowledge, our study is the first to analyse upward exchanges of support during the pandemic and its consequences for the mental health of community-dwelling older Europeans, while acknowledging the role of the pandemic context and the strictness of sanitary measures.

### 2.1 Research question and hypotheses

Intergenerational relations were previously examined in the context of the COVID-19 pandemic (3, 12–14), but very few studies (exception) (27) investigated whether changes in support from adult children to their parents affected the mental health of older adults during these turbulent times. Our main research question is therefore: “Are changes in receiving intergenerational support during the pandemic related to an increase in feelings of sadness and depression?”

We hypothesize that parents who reported an increase in support from adult children during the pandemic are also more likely to experience an increase in depressive feelings, compared to older adults who did not (Hypothesis 1). This hypothesis is based on both pandemic (27) and pre-pandemic (16) studies that already highlighted that increased dependency on family members can have a negative impact on the well-being of older adults.

Nevertheless, we also acknowledge the fact that the pandemic potentially made it more difficult to maintain intergenerational in-person contacts, due to sanitary policies. A sudden drop in support could lead to the older parent’s needs not being met or conflicting expectations about within-family support, and thereby increasing mental distress (27, 33). Therefore, we expect that those who experience a decrease in received intergenerational support are more likely to have an increase in depressive feelings, compared to those who do not (Hypothesis 2).

Taking into account the context of COVID-19, we ask whether and how the pandemic affects this association between intergenerational support and mental health. Concretely, we propose that as restrictions and excess mortality become more severe, the effect of changes in intergenerational support on mental health will become stronger as well (Hypothesis 3). This comes from the assumption that when the severity of the pandemic increases—measured through either higher stringency or higher mortality—the potential for conflicting expectations and family roles rises accordingly (31), with negative mental health outcomes as a potential consequence (8, 30).

## 3 Data and methods

### 3.1 Study population

We use data from the Survey of Health, Ageing and Retirement in Europe (SHARE) (34, 35). In addition to their regular waves, SHARE conducted two rounds of the SHARE Corona Survey between June and August 2020 (SCS1) and 1 year later between June and August 2021 (SCS2). Utilizing SHARE offers the advantage of having multiple measurements during the pandemic (SCS1 and SCS2), as well as prior to the pandemic (e.g., pre-pandemic mental health). These are often not available in the many non-representative samples and internet studies that were quickly initiated after the onset of COVID-19. Additionally, we make use of the interview dates to capture the exact pandemic situation at the time of the interview. Our sample size includes respondents who participated in SCS1, SCS2 or both, with full information on the variables of interest. This results in 42,567 observation across 24,057 respondents aged 65 and over with at least one adult child living outside the household, across 27 countries: Austria, Belgium, Bulgaria, Croatia, Cyprus, Czech Republic, Denmark, Estonia, Finland, France, Germany, Greece, Hungary, Israel, Italy, Latvia, Lithuania, Luxembourg, Malta, Netherlands, Poland, Romania, Slovakia, Slovenia, Spain, Sweden and Switzerland.

### 3.2 Measures

Our dependent variable measures the change in subjective feelings of sadness and depression during the pandemic. Respondents were asked whether they felt sad or depressed. If so, there was a follow-up questions asking if this was “More often,” “About the same” or “Less often” than before the outbreak (SCS1) or than during the first wave (SCS2). We are interested in mental declines. Therefore, following previous studies (36–38), we combined the info of these two questions, resulting into a binary indicator of a decline in mental well-being taking a value of 1 if respondents reported to feel “more often” sad or depressed and 0 otherwise.

Information on receiving intergenerational support was collected through the question “Since the outbreak of Corona, were you helped by others from outside of home to obtain necessities, e.g., food, medications or emergency household repairs?” in SCS1. In SCS2, the same question was asked but with the first outbreak as reference period. After this general question, respondents can clarify from whom they received help. For our analyses, we focus on changes in support provided by non-coresident adult children. The amount of received support is asked with options including “More often,” “Less often” and “About the same.” We classify respondents into three categories distinguishing between (1) those who experienced a decrease (2 and 5% of the total sample for, respectively, SCS1 and SCS2); (2) those who experienced an increase (16 and 9% of the total sample for, respectively, SCS1 and SCS2); (3) those who did not experience a change (5 and 18% of the total sample for, respectively, SCS1 and SCS2), including those who never receive support from their non-coresident children (77 and 68% of the total sample for, respectively, SCS1 and SCS2).

We use two measures from two different data sources in order to capture the pandemic context at the timing of the interview: stringency level and excess mortality. For both measures, it is essential to take into account their high volatility. During the fieldwork of both SCS1 and SCS2, there was a high variability in the stringency level and excess mortality across and within countries as well as during the fieldwork periods of SCS1 and SCS2 (see Supplementary Figures 1, 2). This implies that the timing of the interview is essential to accurately capture the pandemic context (including country-specific policy responses and exposure to COVID-19). Considering these variables merely at the country-level, by for example taking a weighted average, neglects these fluctuations and will not reflect the pandemic context of the respondent at the time of interview. Furthermore, lockdown measures are often implemented overnight and this “shock effect” is likely to affect the mental health of the older population, as well as changes in intergenerational support. It is therefore essential to consider the pandemic context for each individual as accurately as possible. With the availability of the interview dates, we are able to do so. Interviews are distributed across 119 interview dates in SCS1 (June–September 2020) and 75 interview days (June–August 2021) for SCS2 (for distribution of interviews across interview dates: see Supplementary Figure 3).

For the stringency level, we link individual interview dates with data from the Oxford COVID-19 Government Response Tracker (OxCGRT) (39), which tracked daily country-specific governmental policy response to the COVID-19 pandemic. These responses include school closures, workplace closures, cancelation of public events; restrictions on public gatherings; closures of public transport; stay-at-home requirements; public information campaigns; restrictions on internal movements; and international travel controls (39). The OxCGRT data is used to calculate the Oxford Stringency Index, yielding scores from 0 (no restrictions) to 100 (complete lockdown). For this study, we computed a Stringency Index score at the individual level reflecting the mean value of the Stringency Index level between the date of interview and 3 months before.

To account for the levels of exposure to COVID-19, we included information on excess mortality at the time of the respondent’s interview (40). The monthly excess mortality indicator is expressed as the percentage rate of additional deaths in a month, compared to a baseline period. Compared to other measures of exposure to COVID-19 (e.g., confirmed COVID-19 cases), excess mortality has the advantage of avoiding issues of misreporting caused by geographical discrepancies in reporting and testing of COVID-19 (41).

Several potential confounders, known to be associated with intergenerational support and mental health, are included in the analyses. We control for age, gender, number of children and region (Northern Europe, Southern Europe, Western Europe, Eastern Europe and Baltic states). Socio-economic characteristics are based on pre-pandemic data and include the subjective financial situation (i.e., whether the respondent reported that their household is able to make ends meet with great or some difficulty) and level of education. We also controlled for pre-pandemic physical and mental health: pre-pandemic limitations with instrumental activities of daily living (IADL), pre-pandemic limitations with activities of daily living (ADL) and pre-pandemic chronic diseases. For mental health, we control for whether they experienced depression before the pandemic or not. Additionally, we included a measure of subjective health change to indicate a change in health status during the first and/or second wave (i.e., improved health or no change and worsened health). The main characteristics of our analytic sample are summarized in Supplementary Table 1.

### 3.3 Statistical analysis

Given the hierarchical nature of our data, with observations nested within individuals (survey year) which are in turn nested within countries, we conduct multilevel logistic modeling. Although our models do not contain variables measured at the country-level, multilevel modeling ensures a more accurate parameter estimation by accounting for the hierarchical nature of our data, compared to models that assume independence between observations (42). In a robustness check, we ignore the hierarchical structure of our dataset and repeat the analyses by performing a pooled OLS regressions with clustered standard errors to account for within-person correlation and country-fixed effects (see Supplementary material). We start our main analyses with a null model to assess the extent of the cross-country and temporal variability of our dependent variable, by only including the fixed intercept, and the country-level and survey year-level (SCS1 or SCS2) random intercept variances. Intra-class correlation coefficients (ICC) are estimated to assess these variance components. We then proceed by examining whether changes in intergenerational support affects the likelihood of experiencing increased depressive feelings, while controlling for relevant covariates (Model 1). Model 2 includes measures of pandemic context, i.e., stringency index and excess mortality. We stress once again that these measures of pandemic context are included at the individual-level, since they are linked to each respondent’s date of interview to capture the measures of pandemic context with the concurrent level of depressive feelings and intergenerational support. Lastly, interaction terms are added in order to explore differences in the effect of changes of intergenerational support by variables related to the pandemic context. For this, interactions with the stringency index (Model 3) and excess mortality (Model 4) are first added separately, before considering them simultaneously (Model 5). Stata code to replicate the analyses are available online.

We perform additional robustness checks to eliminate potential bias in our results. First, we test whether our results are specific for the pandemic or whether similar mechanisms are found when studying pre-pandemic data. Second, we exclude measures of changes in self-rated health in order to account for the potential overlap between the assessment of overall health and mental health. Finally, we repeat our analyses with an additional category in our measurement of intergenerational support, in order to differentiate between respondents receiving no support and respondents experiencing no change in support (see Supplementary material).

## 4 Results

### 4.1 Mental health during the pandemic

At the start of the pandemic, 17% of our respondents had more often depressive feelings after the first outbreak compared to before. Across all included countries, the share ranges from 9% (Denmark) to 31% (Spain). We observe the highest proportion of respondents with increased depressive feelings in Southern Europe (19%) and the lowest proportion in Northern Europe (12%) (see SCS1 in Supplementary Figure 4).

As COVID-19 continued to rage across Europe, 12% of the respondents reported a worsening of mental health after the second outbreak, compared to the first outbreak. During the second outbreak, the Baltic States reported the highest proportion (15%) of increased depressive feelings, while the lowest proportion was observed in Northern Europe (6%) (see SCS2 in Supplementary Figure 4). Although the proportion of individuals reporting a worsening of their mental health is highest after the first outbreak, we see that the pandemic continues to impact the mental health of older Europeans also after the first wave of infections. This is especially true for the Baltic states and Eastern Europe, where we do not observe a decline in the share of respondents indicating worsening mental health.

### 4.2 Intergenerational support during the pandemic

Looking at changes in receiving intergenerational support across Europe (see SCS1 in Supplementary Figure 5), it seems that the family took up the role of caregiver more intensively as a response to the first outbreak of COVID-19. This trend was noticeable all over Europe with differences across regions being rather limited. Increases in intergenerational support during the first wave ranges between 18% in the Northern countries and 26% in the Western countries. In parallel, between 5% (in Southern Europe) and 4% (in Northern Europe) of respondents indicate a decrease in intergenerational support. As the pandemic continued some discrepancies between geographical regions emerged. These descriptives show that increases in support between parents and their adult children were more prominent in Southern Europe (13%), Eastern Europe (13%) and the Baltic States (12%), compared to Northern (4%) and Western Europe (7%) (see SCS2 in Supplementary Figure 5).

### 4.3 Effect of changes in intergenerational support on depressive feelings

In what follows, we explore whether and to what extent changes in receiving intergenerational support affected the mental health of older parents during the pandemic. The null model (See Supplementary Table 2) and its corresponding ICCs show that about 3% of the variability in experiencing increased depressive feelings is explained by between-country differences, and about 35% lies between time points within countries. It are thus primarily individual differences and temporal differences that account for the variation in increased depressive feelings, rather than differences across countries.

Model 1 (see Table 1) adds measures for change in intergenerational support, adjusted for potential confounding of pre-and intra-pandemic socio-economic characteristics, health and depression. Receiving more support from one’s children during the pandemic is significantly related to a higher likelihood of experiencing an increase in depressive feelings, compared to those who maintained the same level of intergenerational support. In contrast, those who experienced a decrease in intergenerational support were not significantly more or less likely to experience an increase in mental distress. These results are in line with hypothesis 1 but not with hypothesis 2.

**Table 1:** Multilevel logistic modelling results (expressed in odds ratios) on increased depressive feelings.

	Model 1		Model 2		Model 3		Model 4		Model 5	
	OR	95% CI	OR	95% CI	OR	95% CI	OR	95% CI	OR	95% CI
*Fixed part*										
Intercept	0.03***	(0.02–0.04)	0.02***	(0.01–0.03)	0.02***	(0.01–0.03)	0.02***	(0.01–0.03)	0.02***	(0.01–0.04)
Age 75+ (ref. 65–74 years old)	0.99	(0.92–1.06)	0.98	(0.92–1.06)	0.98	(0.91–1.06)	0.98	(0.91–1.06)	0.98	(0.91–1.06)
IADL limitations (ref. no IADL limitations)	1.01	(0.92–1.11)	1.01	(0.91–1.11)	1.01	(0.91–1.11)	1.00	(0.91–1.11)	1.00	(0.91–1.10)
ADL limitations (ref. no ADL limitations)	1.07	(0.95–1.19)	1.07	(0.95–1.19)	1.07	(0.95–1.19)	1.06	(0.95–1.19)	1.06	(0.95–1.19)
Chronic diseases (ref. no chronic diseases)	1.24***	(1.15–1.33)	1.24***	(1.15–1.33)	1.24***	(1.15–1.34)	1.24***	(1.15–1.34)	1.24***	(1.15–1.34)
Female (ref. male)	1.69***	(1.57–1.83)	1.69***	(1.57–1.83)	1.69***	(1.57–1.83)	1.70***	(1.57–1.83)	1.70***	(1.57–1.83)
Financial difficulties (ref. no)	1.31***	(1.21–1.42)	1.31***	(1.20–1.42)	1.31***	(1.20–1.42)	1.31***	(1.20–1.42)	1.31***	(1.20–1.42)
More than one child (ref. one child)	0.91**	(0.84–1.00)	0.91**	(0.84–0.99)	0.91**	(0.84–0.99)	0.92**	(0.84–1.00)	0.91**	(0.84–1.00)
Decline in health (ref. maintains health status)	4.68***	(4.29–5.11)	4.69***	(4.30–5.12)	4.69***	(4.29–5.12)	4.68***	(4.28–5.11)	4.67***	(4.28–5.10)
Depressed before first outbreak (ref. no)	2.35***	(2.18–2.54)	2.35***	(2.18–2.55)	2.36***	(2.18–2.55)	2.36***	(2.18–2.55)	2.36***	(2.18–2.55)
No partner in household (ref. with partner)	1.13***	(1.05–1.22)	1.13***	(1.05–1.22)	1.13***	(1.05–1.22)	1.13***	(1.04–1.22)	1.13***	(1.04–1.22)
Educational level (ref. ISCED 0–2)										
ISCED 3–4	0.98	(0.90–1.07)	0.98	(0.90–1.06)	0.98	(0.90–1.06)	0.98	(0.90–1.06)	0.98	(0.90–1.07)
ISCED 5–6	1.12**	(1.01–1.24)	1.12**	(1.01–1.24)	1.12**	(1.01–1.24)	1.12**	(1.01–1.24)	1.12**	(1.01–1.24)
European region (ref. Northern Europe)										
Western Europe	1.57**	(1.04–2.37)	1.46*	(0.99–2.15)	1.46*	(0.99–2.16)	1.47*	(1.00–2.18)	1.48**	(1.00–2.19)
Southern Europe	1.57**	(1.04–2.36)	1.48*	(1.00–2.20)	1.47*	(0.99–2.19)	1.49**	(1.00–2.22)	1.49*	(1.00–2.22)
Eastern Europe	1.16	(0.76–1.77)	1.12	(0.75–1.67)	1.11	(0.74–1.67)	1.13	(0.75–1.69)	1.13	(0.75–1.69)
Baltic states	1.16	(0.71–1.89)	1.24	(0.78–1.96)	1.23	(0.77–1.95)	1.23	(0.77–1.95)	1.22	(0.77–1.95)
Time: second outbreak (ref. first outbreak)	0.66***	(0.61–0.70)	0.73***	(0.67–0.80)	0.73***	(0.67–0.80)	0.72***	(0.66–0.79)	0.72***	(0.66–0.79)
Intergenerational support (ref. no change)										
Less often	1.11	(0.95–1.29)	1.11	(0.95–1.29)	0.23**	(0.07–0.79)	1.30***	(1.07–1.57)	0.30*	(0.09–1.08)
More often	1.82***	(1.68–1.98)	1.82***	(1.68–1.98)	1.74*	(0.91–3.31)	1.63***	(1.47–1.81)	1.14	(0.57–2.25)
Stringency index at time of interview			1.01***	(1.00–1.02)	1.01**	(1.00–1.02)	1.01***	(1.00–1.02)	1.01*	(1.00–1.01)
Excess mortality at time of interview			0.99**	(0.98–1.00)	0.99**	(0.98–1.00)	0.99***	(0.98–1.00)	0.99***	(0.98–1.00)
Stringency index * intergenerational support										
Less often					1.02**	(1.01–1.04)			1.02**	(1.00–1.04)
More often					1.00	(0.99–1.01)			1.01	(1.00–1.02)
Excess mortality * intergenerational support										
Less often							0.96**	(0.94–0.99)	0.97**	(0.94–1.00)
More often							1.02***	(1.01–1.03)	1.02***	(1.01–1.04)
*Random part—variance parameters*										
Between-country variance	0.08	(0.04–0.15)	0.07	(0.03–0.13)	0.07	(0.03–0.13)	0.07	(0.03–0.13)	0.07	(0.04–0.14)
Between-time variance	1.00	(0.83–1.20)	1.00	(0.83–1.20)	1.00	(0.83–1.20)	1.00	(0.83–1.20)	1.00	(0.81–1.21)
-2 Log Likelihood	15,420.7		15,413.8		15,410.5		15,403.0		15,400.1	

95% CI in parentheses; ***p < 0.01, **p < 0.05, *p < 0.1.

Looking at our covariates, the results also indicate important gender differences. Women were more likely to suffer from increased depressive feelings during the pandemic compared to men. Furthermore, those who were depressed before the pandemic, lived without a partner, experienced a decline in subjective physical health, and had financial difficulties were all more likely to experience an increase in depressive feelings during the pandemic. Across geographical regions, we see that respondents living in Western parts of Europe reported a higher likelihood of increased depressive feelings, compared to those living in Northern Europe. Respondents were less likely to report an increase in depressive feelings during SCS2, compared to SCS1.

In the following models, we include more specific measures of the pandemic context, i.e., stringency index and excess mortality at the time of interview. We find a positive association between increased depressive feelings and higher scores on the stringency index, and a negative association with excess mortality (Model 2 in Table 1). While lockdowns, social distancing and other restricting guidelines have significant negative mental health consequences, the pandemic can also give rise to feelings of unity, resilience, and sense of belonging, and thereby buffer against depressive symptoms.

**Figure 1:**
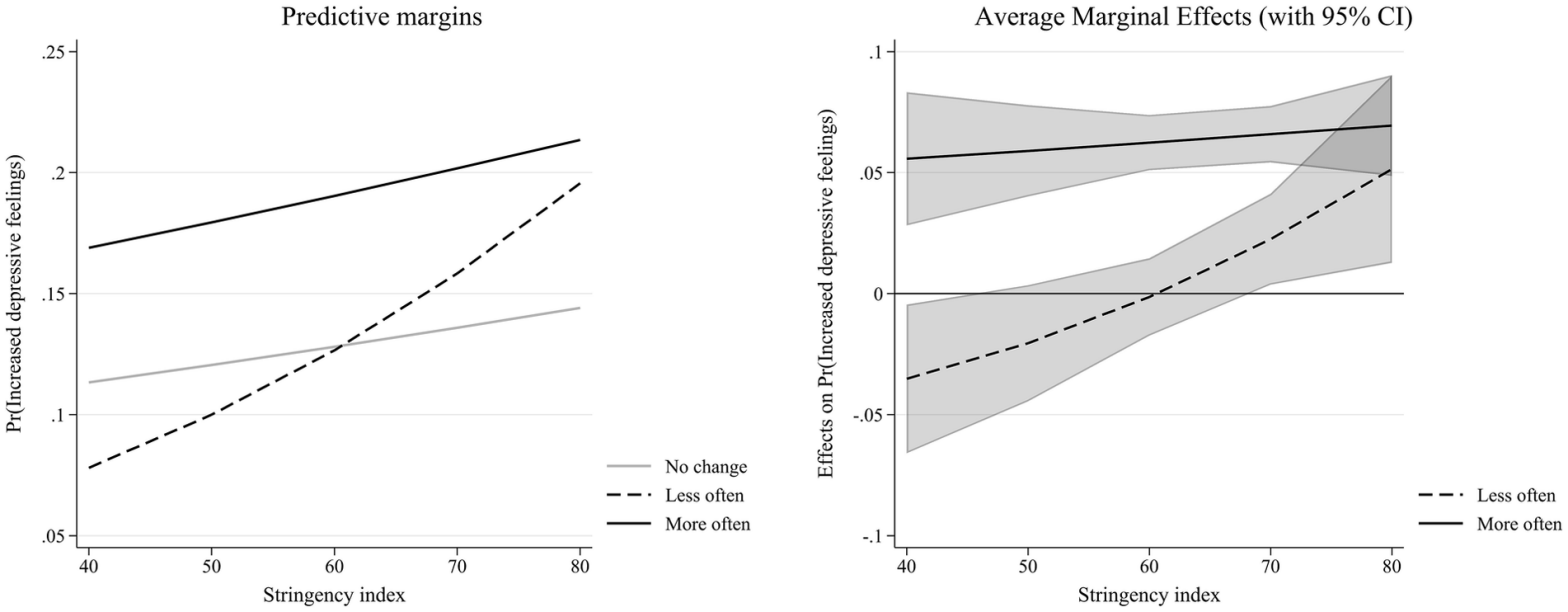
Interaction effect between changes in intergenerational support and stringency index (Table 1—Model 3).

Additionally, the effects of changes in intergenerational support depend on the level of these two measures of pandemic context, which is partially in line with hypothesis 3 (see Model 3 in Table 1). While the effect of more intergenerational support does not depend on the level of stringency, the effect of decreases in received intergenerational support does. Experiencing a decline in received support is related to a lower likelihood of increased depressive feelings but this negative effect is attenuated as more stringent measures are in force. Declines in support are potentially direct responses to high stringency levels, which might be detrimental for older adult’s mental health. Visually, we see that the slopes of experiencing no change and increases in intergenerational support are quite similar, while the slope of decreases in support is much steeper (see left panel of Figure 1). Indeed, the difference between those that are experiencing no change and those that report an increase in support remains continuously stable as stringency increases (see right panel of Figure 1). The difference between no change (i.e., the reference category) and decreases in support, however, increases with stringency and becomes significant when the stringency level exceeds a value of approximately 65 (see right panel of Figure 1).

**Figure 2:**
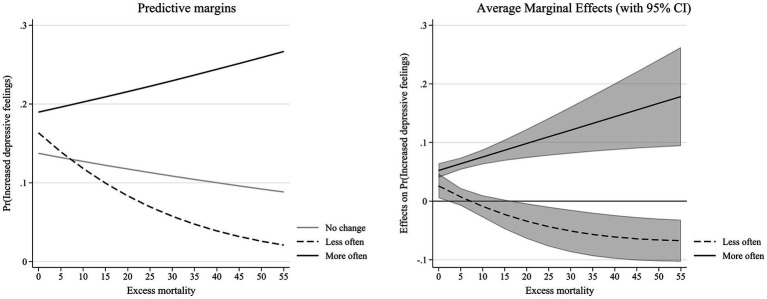
Interaction effect between changes in intergenerational support and excess mortality (Table 1—Model 4).

Turning to excess mortality, a different pattern emerges (see Model 4 in Table 1). We now observe a positive effect of receiving more support, and this effect increases with higher excess mortality, visualized by a positive slope (see left panel of Figure 2). Receiving less support is now related to higher likelihood of increased depressive feelings. This effect, however, decreases as excess mortality rises (see right panel of Figure 2). For lesser support, the average marginal effect becomes more negative with mortality but the difference is only significant for high levels of excess mortality (see right panel of Figure 2).

Our full model (see Model 5 in Table 1), including both interactions between pandemic context and changes in intergenerational support, confirms these results. Again, we see that the effect of decreases in support depends on the level of stringency, with a higher likelihood of increased depressive feelings as stringency rises. For excess mortality, increases in support are related to higher likelihood of increasing depressive feelings as excess mortality reaches higher levels. Decreases in support, on the contrary, lower the likelihood with rising excess mortality.

## 5 Discussion

An extensive body of research has looked into the mental health and well-being consequences of the pandemic, with a range of studies focusing on the older population (18, 29, 43, 44). Nevertheless, the mechanisms behind potential mental well-being effects are far less illuminated. In this study, we examined the relationship between changes in intergenerational support and experiencing an increase in depressive feelings among the 65+ population. In doing so, we cover the COVID-19 pandemic from its initial outbreak until the Summer of 2021 and thereby provide a comprehensive assessment of its impact.

We find that about 17% of individuals aged 65 and older reported feeling more sad or depressed during the first outbreak compared to before. This number decreases to 13% when comparing SCS2 with SCS1. At the same time, we also see a drastic increase in intergenerational support after the onset of the pandemic. Although social interactions were discouraged or even prohibited, many families maintained or even increased their support exchanges. Previous studies showed that older adults’ level of intergenerational interactions were more likely to increase in countries with more stringent measures (14). These increases in support are likely responses to intensified pre-existing needs for support as well as newly created needs among older adults who were previously not relying on external support (45).

With limited formal alternatives, the cost of exchanges between parents and adult children are likely to be higher than under normal circumstances. Family caregivers may be feeling obligated to fill in the care void or decide that the exchange is not worth the risk. Both scenarios may translate into ambivalence, tension between caregiver and care-recipient and increased mental distress for both parties (22).

Our first two research hypotheses therefore examined whether these changes in receiving intergenerational support during to the pandemic were related to deteriorating mental well-being among 65+ Europeans. We found that older individuals who experienced an increase in received support during the pandemic were more likely to report an increase in depressive feelings, compared to those who did not experience a change in intergenerational support. This positive association between increases in intergenerational support and increases in depressive feelings may not only reflect the negative effects of increased dependence, loss of self-efficacy and control (16, 25), but also resonates with theories emphasizing the importance of reciprocity in intergenerational exchanges in order to produce positive mental health outcomes (46). The balance between giving and receiving may become distorted due to the pandemic, as it limits all kinds of support exchanges (e.g., grandchild care), making it difficult for older parents to fulfill this reciprocity condition. Nevertheless, it could also be the case that respondents who experienced an increase in depressive feelings, called upon their support network more often, resulting in increasing intergenerational support. For decreases in support, no significant effects were found when the pandemic context was not taken into account.

Our third research hypothesis focused on the role of the epidemiological situation. Given the vast differences in the strictness of the sanitary measures as well as in the severeness of the pandemic, it is crucial to relate concurrent pandemic measures to both changes in intergenerational exchanges and mental health. Stringency levels and excess mortality are found to be, respectively, positively and negatively related to increased depressive feelings. This contrasting finding highlights the complex interplay between individual experiences and societal responses to crises. While the pandemic can cause strain and challenges caused by social distancing and other measures, it can also be a source of community and resilience. This shows that socially disruptive events are not necessarily always related to lower well-being for all (47).

Furthermore, we find that the effect of decreases in intergenerational support actually depends on the level of stringency. The positive effect of less support increases as more stringency measures are implemented - but only when it surpasses a certain level. A possible interpretation for this is that when lower levels of support are not solely an autonomous decision of the family but also urged by sanitary policies. When decisions on intergenerational support are not self-determined, but potentially induced by restricting policies, constraints and fear, this might lead to worse mental health outcomes. Additionally, it may create intergenerational tension. As adherence to these measures might differ between adult children and their parents (31), expectations about support might not be in concordance as well. Greater stringency also prevents other sources of social contact and informal support (e.g., other family members, friends and neighbors) as well as formal care alternatives, which are otherwise potential substitutes for intergenerational contacts. When older parents are left with unmet needs or expectations, this may not only impact their physical but also mental well-being (33). This finding signals the importance of the family as source of support and well-being. But the causality can run the other way around as well: older parents experiencing worse mental health due to higher stringency levels, may become socially isolated and in turn limit their intergenerational contacts (48). Based upon our results, we cannot fully assess the directionally or existence of causality between decreases in intergenerational support and late-life well-being.

Such association, however, is not found for increases in intergenerational support with the detrimental effect of increases support on mental health being consistent across different levels of stringency. The fact that adult children may intensify their role as caregiver does not seem to interfere with the connection between changes in intergenerational support and mental health. For increasing mortality, on the other hand, we see that it is related to stronger effects of increases in intergenerational support on mental health. When COVID-19 related deaths are high, this could lead to increased fear and anxiety for getting infected when receiving in-person support. The effect of decreases in intergenerational support, however, decreases with mortality rates. Respondents might argue that such decreases are necessary in the concurrent context which thereby decreases its negative mental health impact.

Interpretation of these results should take account of the limitations of the study. Findings are specific to older Europeans and exchanges of support with adult children. Therefore these are not generalisable to non-European contexts, younger age groups or support exchanged with other relatives or non-family members. Due to the question wording, we were not able to differentiate between different types and intensities of support. Potential mental health effects of, for example, emotional support should be addressed in future research. Additionally, our measure of mental health is captured by a single item instead of a composite depression scale. Our dependent measure should therefore be interpreted as a subjective indicator of increase in depressive feelings that suffers from potential recall bias. An interesting pathway for future research may be to explore whether similar mechanisms are found when looking at measures of changes in positive affect (e.g., increase in life satisfaction). Such measures, unfortunately, were not part of SCS1 nor SCS2. Furthermore, the fact that changes in both intergenerational support and mental health are based upon the subjective evaluation of the respondent, makes that our results cannot be interpreted as causal. For this, again, a composite depression scale, measured at different time-points would reduce this limitation to some extent, but is unfortunately not available for the European older population during the pandemic. Our results also confirm prior research examining whether and how the pandemic’s effects vary by gender. Women are found to be disproportionately affected by containment and closure policies when looking at mental distress outcomes (36). In our sample, women indeed report higher levels of depressive feelings compared to men. Forthcoming studies should build upon these results and disentangle whether these gender differences originate directly from containment policies or indirectly through, for example, changes in informal care and support. Previous research already showed that men are more likely to report decreased parent–child contacts than women during the pandemic (14). Whether these gender differences also translate into diverging mental health outcomes is still unknown.

To conclude, our study demonstrates the relevance of the family and intergenerational relations for the mental health of the older generation during an unprecedented time that is the COVID-19 crisis. While receiving adequate support is essential for late-life well-being, we should acknowledge that intergenerational exchanges of care and support are also potential stress factors and sources of ambivalence, not only for the care provider but also for the recipient. When family relations are put under pressure, like during a global pandemic, this might translate into poorer mental health outcomes. Formal care alternatives should complement informal support in order to limit potential conflict in expectations and family roles. This is not only relevant for future policies designed to combat the spread of infectious diseases similar to COVID-19, but also in the context of population ageing and its related increase in healthcare costs, where the family functions as an important provider of informal care (49). Social policies targeting older adults living at home should take these consequential effects of intergenerational support into careful consideration as well, as good family ties are vital for late-life well-being.

## Data Availability

Publicly available datasets were analyzed in this study. This data can be found at: https://share-eric.eu.
