# Corrigendum: The family as provider of intergenerational support during COVID-19: a study into the mental health consequences for 65+ Europeans

**DOI:** 10.3389/fpubh.2024.1530317

**Published:** 2024-12-10

**Authors:** Lore Van Herreweghe, Wim Van Lancker

**Affiliations:** Centre for Sociological Research, University of Leuven, Leuven, Belgium

**Keywords:** intergenerational support, intergenerational relations, informal care, mental health, COVID-19

In the published article, there was an error in the captions of Figures 1, 2 as published. The correct captions of Figures 1, 2 appear below.

**Figure 1:**
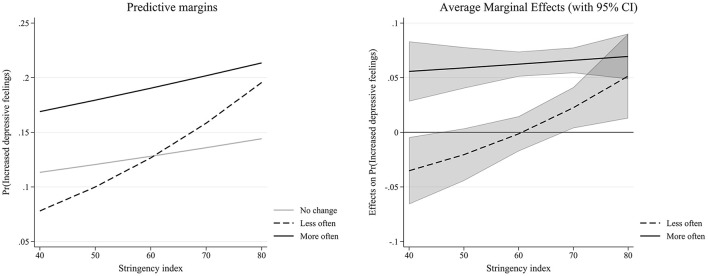
Interaction effect between changes in intergenerational support and stringency index (Table 1—Model 3).

**Figure 2:**
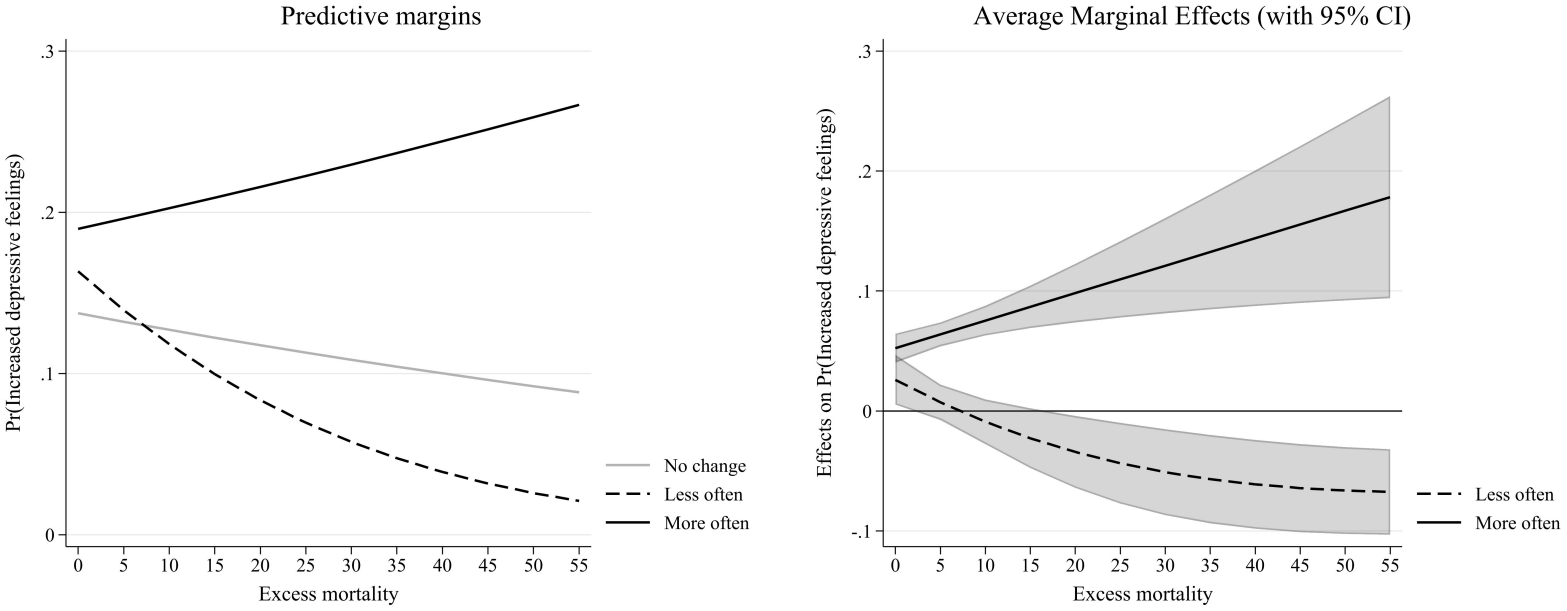
Interaction effect between changes in intergenerational support and excess mortality (Table 1—Model 4).

In the published article, Supplementary Figures 4, 5 were mistakenly not included in the publication. The missing figures, Supplementary Figures 4, 5, are included in the corrected version of the Supplementary Material.

In the published article, there was an error in the text. The text in **4.1. Mental health during the pandemic** incorrectly cited Figure 1 when Supplementary Figure 4 should be cited. A correction has therefore been made to **4.1. Mental health during the pandemic**.

The text previously stated:

“At the start of the pandemic, 16% of our respondents had more often depressive feelings after the first outbreak compared to before. Across all included countries, the share ranges from 9% (Denmark) to 31% (Spain). We observe the highest proportion of respondents with increased depressive feelings in Southern Europe (19%) and the lowest proportion in Northern Europe (12%) (see SCS1 in Figure 1).

As COVID-19 continued to rage across Europe, 12% of the respondents reported a worsening of mental health after the second outbreak, compared to the first outbreak. During the second outbreak, the Baltic states reported the highest proportion (15%) of increased depressive feelings, while the lowest proportion was observed in Northern Europe (6%) (see SCS2 in Figure 1). Although the proportion of individuals reporting a worsening of their mental health is highest after the first outbreak, we see that the pandemic continues to impact the mental health of older Europeans also after the first wave of infections. This is especially true for the Baltic states and Eastern Europe, where we do not observe a decline in the share of respondents indicating worsening mental health.”

The corrected text appears below:

“At the start of the pandemic, 17% of our respondents had more often depressive feelings after the first outbreak compared to before. Across all included countries, the share ranges from 9% (Denmark) to 31% (Spain). We observe the highest proportion of respondents with increased depressive feelings in Southern Europe (19%) and the lowest proportion in Northern Europe (12%) (see SCS1 in Supplementary Figure 4).

As COVID-19 continued to rage across Europe, 12% of the respondents reported a worsening of mental health after the second outbreak, compared to the first outbreak. During the second outbreak, the Baltic states reported the highest proportion (15%) of increased depressive feelings, while the lowest proportion was observed in Northern Europe (6%) (see SCS2 in Supplementary Figure 4). Although the proportion of individuals reporting a worsening of their mental health is highest after the first outbreak, we see that the pandemic continues to impact the mental health of older Europeans also after the first wave of infections. This is especially true for the Baltic states and Eastern Europe, where we do not observe a decline in the share of respondents indicating worsening mental health.”

In the published article, there was an error in the text. The text in **4.2. Intergenerational support during the pandemic** incorrectly cited Figure 2 when Supplementary Figure 5 should be cited. A correction has therefore been made to **4.2. Intergenerational support during the pandemic**.

The text previously stated:

“Looking at changes in receiving intergenerational support across Europe (see SCS1 in Figure 2), it seems that the family took up the role of caregiver more intensively as a response to the first outbreak of COVID-19. This trend was noticeable all over Europe with differences across regions being rather limited. Increases in intergenerational support during the first wave ranges between 18% in the Northern countries and 26% in the Western countries. In parallel, between 5% (in Southern Europe) and 4% (in Northern Europe) of respondents indicate a decrease in intergenerational support. As the pandemic continued some discrepancies between geographical regions emerged. These descriptives show that increases in support between parents and their adult children were more prominent in Southern Europe (13%), Eastern Europe (13%) and the Baltic States (12%), compared to Northern (4%) and Western Europe (7%).”

The corrected text appears below:

“Looking at changes in receiving intergenerational support across Europe (see SCS1 in Supplementary Figure 5), it seems that the family took up the role of caregiver more intensively as a response to the first outbreak of COVID-19. This trend was noticeable all over Europe with differences across regions being rather limited. Increases in intergenerational support during the first wave ranges between 18% in the Northern countries and 26% in the Western countries. In parallel, between 5% (in Southern Europe) and 4% (in Northern Europe) of respondents indicate a decrease in intergenerational support. As the pandemic continued some discrepancies between geographical regions emerged. These descriptives show that increases in support between parents and their adult children were more prominent in Southern Europe (13%), Eastern Europe (13%) and the Baltic States (12%), compared to Northern (4%) and Western Europe (7%) (see SCS2 in Supplementary Figure 5).”

The authors apologize for these errors and state that this does not change the scientific conclusions of the article in any way. The original article has been updated.

