# Supplementary material for: The family as provider of intergenerational support during COVID-19: a study into the mental health consequences for 65+ Europeans
Source: Front Public Health. 2024 Oct 21;12:1418472. doi: 10.3389/fpubh.2024.1418472 (PMC11532062; doi:10.3389/fpubh.2024.1418472)
Supplement: Supplementary file 1 [file Data_Sheet_1.pdf]

Supplementary Material

1 Pandemic context

We use two measures from two different data sources in order to capture the pandemic context at the timing of the interview: stringency level and excess mortality. For both measures, it is essential to take into account their high volatility. During the fieldwork of both SCS1 and SCS2, there was a high variability in the stringency level and excess mortality across and within countries (see Figures S1 and S2). This implies that the timing of the interview is essential to accurately capture the pandemic context. Considering these variables merely at the country-level, by for example taking a weighted average, neglects these fluctuations and will not reflect the pandemic context of the respondent at the time of interview. Furthermore, lockdown measures are often implemented overnight and this 'shock effect' is likely to affect the mental health of the older population, as well as changes in intergenerational support. It is therefore essential to consider the pandemic context for each individual as accurately as possible. With the availability of the interview dates, we are able to do so. Interviews are distributed across 119 interview dates in SCS1 (June - September 2020) and 75 interview days (June - August 2021) for SCS2 (for distribution of interviews across interview dates: see Figure S3).

For the stringency level, we link individual interview dates with data from the Oxford COVID-19 Government Response Tracker (OxCGRT) (Hale et al., 2021), which tracked daily country-specific governmental policy response to the COVID-19 pandemic. (Hale et al., 2021). These responses include school closures, workplace closures, cancellation of public events; restrictions on public gatherings; closures of public transport; stay-at-home requirements; public information campaigns; restrictions on internal movements; and international travel controls. The OxCGRT data is used to calculate the Oxford Stringency Index, yielding scores from 0 (no restrictions) to 100 (complete lockdown). For this study, we computed a Stringency Index score at the individual level reflecting the mean value of the Stringency Index level between the date of interview and three months before.

To account for the levels of exposure to COVID-19, we included information on excess mortality at the time of the respondent's interview (Eurostat, 2022). The monthly excess mortality indicator is expressed as the percentage rate of additional deaths in a month, compared to a baseline period. Compared to other measures of exposure to COVID-19 (e.g., confirmed COVID-19 cases), excess mortality has the advantage of avoiding issues of misreporting caused by geographical discrepancies in reporting and testing of COVID-19 (Beaney et al., 2020).

2 Statistical analyses: multilevel modelling

To assess the extent of the cross-country and temporal variability of our dependent variable, we start with a null model, only including the fixed intercept, and the country-level and survey year-level (SCS1 or SCS2) random intercept variances. Intra-class correlation coefficients (ICC) are estimated to assess these variance components. We then proceed by examining whether changes in intergenerational support affects the likelihood of experiencing increased depressive feelings, while controlling for relevant covariates (Model 1). Model 2 includes measures of pandemic context, i.e. stringency index and excess mortality. It is important to note that these measures of pandemic context are included at the individual-level, since they are linked to each respondent's date of interview to

capture the measures of pandemic context with the concurrent level of depressive feelings and intergenerational support. Lastly, interaction terms are added in order to explore differences in the effect of changes of intergenerational support by variables related to the pandemic context. For this, interactions with the stringency index (Model 3) and excess mortality (Model 4) are first added separately, before considering them simultaneously (Model 5). Stata code to replicate the analyses are available online.

3 Robustness checks

We perform several robustness checks to eliminate potential bias in our results. First, we test whether our results are specific for the pandemic or whether similar mechanisms are found when studying pre-pandemic data. Second, we ignore the hierarchical structure of our dataset and repeat the analyses by performing a pooled OLS regressions. We will here apply clustered standard errors to account for within-person correlation and add country-fixed effects. Third, we exclude measures of changes in self-rated health in order to account for the potential overlap between the assessment of overall health and mental health. Finally, we repeat our analyses with an additional category in our measurement of intergenerational support, in order to differentiate between respondents receiving no support and respondents experiencing no change in support.

3.1 Results

As a first robustness check, we assessed whether the link between intergenerational support and mental health also applies to pre-pandemic times - and may thus be valid in general - or only in the specific context of the pandemic. When we estimate a similar model but now using measures of change in intergenerational support and mental health captured between 2013 and 2015 (i.e. SHARE wave 5 and wave 6; wave 7 was not used due to the SHARELIFE survey which focuses on respondents' life histories), we see that increases in intergenerational support are also related to increases in depressive symptoms (measured by means of increases on EURO-D scale). We do not, however, find support for decreases in intergenerational support being associated with changes in depressive feelings (see Table S3). The fact that increases in support potentially trigger feelings of dependency and losing autonomy, makes that more support is detrimental for older adults' mental health, irrespective of the global health situation. When older adults' experience a decrease in informal support, however, context does matter. When this decrease is a response to policy restrictions and/or health crises, it might be bad for the mental well-being of the care-recipient. Future research should investigate whether there are other specific conditions under which decreases in support are related to (detrimental) changes mental health. Secondly, we estimated pooled OLS regression with clustered standard errors to account for within-person correlation and add country-fixed effects. These models do not take into account the hierarchical structure of our dataset. Nevertheless, the results are similar to our multilevel models (see Table S4). As already shown by the ICCs of our multilevel models, almost all variance is attributable to the lower levels (i.e. individuals and observation within individuals). Thirdly, we repeated all multilevel analyses but excluded measures of self-rated physical health, since there might be potential overlap. All results were confirmed in these analyses (results not shown). Lastly, we added an additional dummy in order to separate those who did not experience a change in intergenerational support from those who did not receive intergenerational support. Since no differences emerged (results not shown), we pooled the two categories together in the main analyses, to ease interpretation.

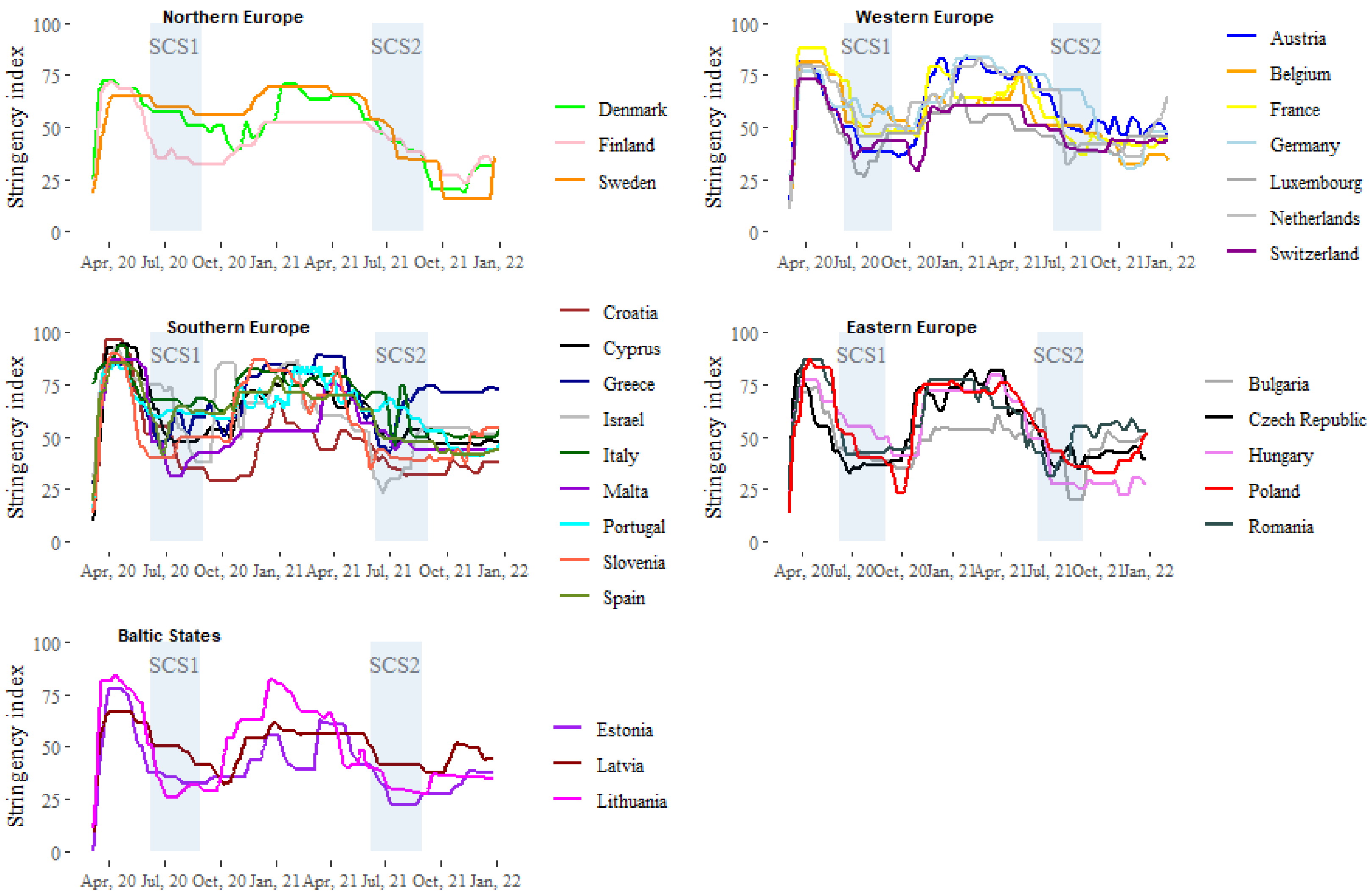

Supplementary Figure 1. Evolution of stringency index across countries

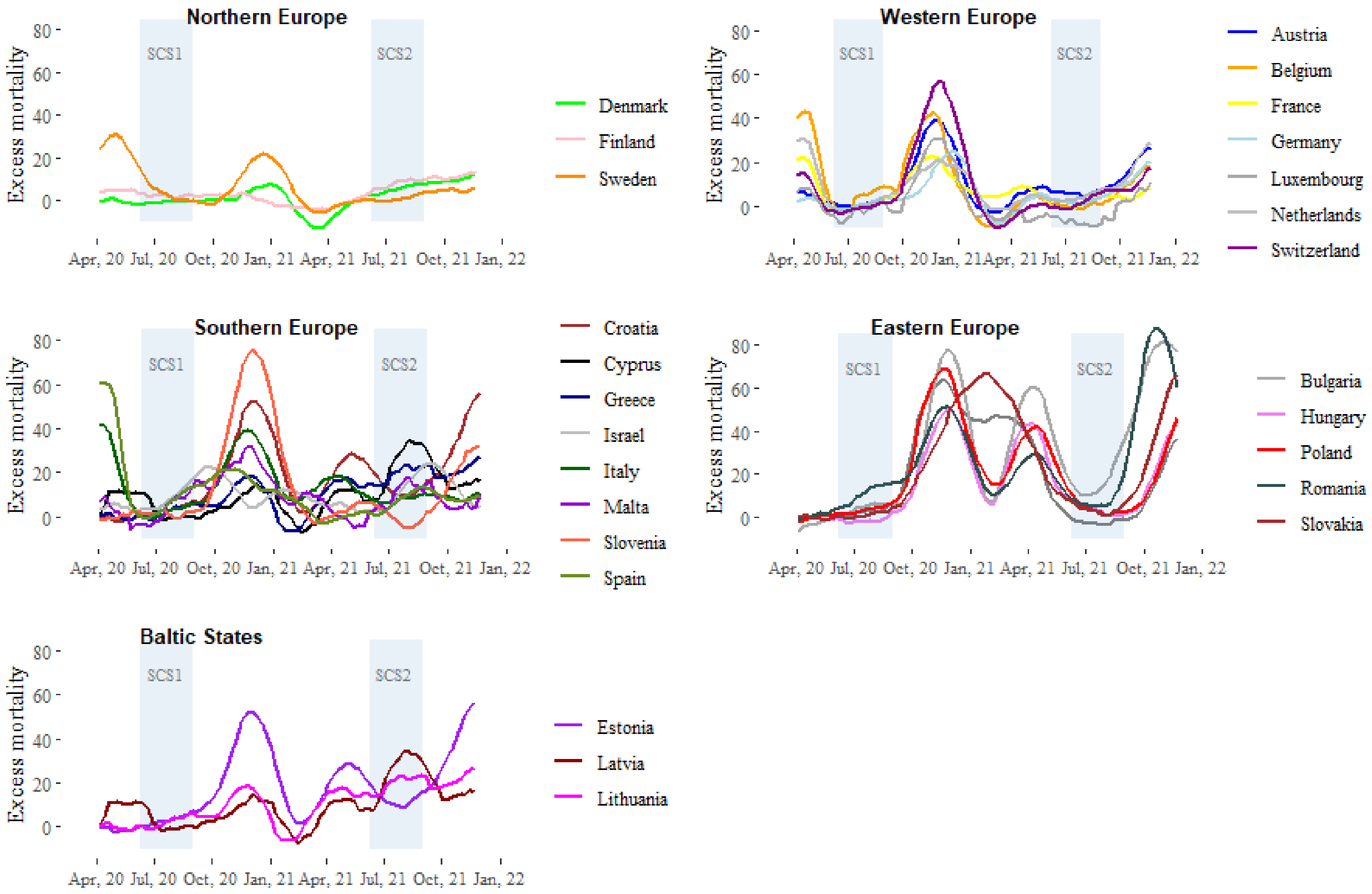

Supplementary Figure 2. Evolution of excess mortality across countries

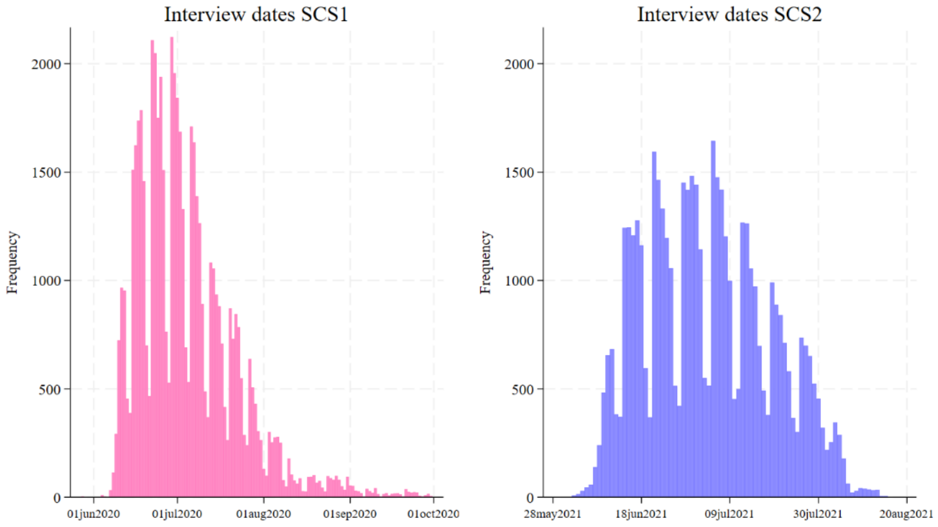

Supplementary Figure 3. Distribution of interviews across interview dates in SCS1 and SCS2

Supplementary Table 1. Descriptive statistics (42,567 observations across 24,057 respondents)

	SCS1		SCS2	
	%	*N*	%	*N*
Age category				
65-74 years old	54	12,124	53	10,746
75 or older	46	10,236	47	9,461
Gender				
Female	57	12,761	58	11,621
Male	43	9,599	42	8,586
Number of children				
One child	20	4,415	19	3,875
Two or more children	80	17,945	81	16,332
Education				
Low (ISCED 0-2)	36	8,149	35	7,125
Medium (ISCED 3-4)	42	9,312	43	8,576
High (ISCED 5-6)	22	4,899	22	4,506
Partner in household				
Yes	67	14,973	67	13,529
No	33	7,387	33	6,678
Financial difficulties				
Yes	32	7,078	30	6,226
No	68	15,282	70	14,092
IADL (pre-pandemic)				
No	80	17,791	82	16,546
Yes	20	4,569	18	3,658
ADL (pre-pandemic)				
No	88	19,726	90	18,124
Yes	12	2,634	10	2,083
Chronic diseases (pre-pandemic)				
Less than 2 diseases	40	8,966	41	11,844
2 or more diseases	60	13,394	59	8,363
Change in health				
Improvement or no change	90	20,200	85	17,233
Worse health	10	2,160	15	2,974
Pre-pandemic depression				
Yes	27	6,100	26	5,267
No	73	16,260	74	14,940
Intergenerational support				
No change or support	74	16,636	84	16,909
Less often	4	797	6	1,208
More often	22	4,927	10	2,091
European region				
Northern	11	2,463	10	2,011
Western	29	6,395	29	5,772
Southern	28	6,369	30	6,398
Eastern	19	4,336	19	3,852
Baltic states	13	2,797	13	2,623

Supplementary Table 2. Multilevel logistic model – null model

	**Model 0**	
	OR	[95% CI]
Fixed part		
Intercept	0.10***	(0.08 - 0.12)
Random part		
Variance parameters		
Between-country variance	0.15	(0.08 – 0.26)
Between-time variance	1.64	(1.43 – 1.87)
-2 Log Likelihood	-17,118.96	

Notes: *p < 0.05; **p < 0.01; ***p < 0.001; OR: odds ratio; SE: standard errors

Supplementary Table 3. Pre-pandemic robustness check - Logistic regression results (Odds ratios) on increased scores on EURO-D scale between 2013 and 2015.

	Model 5	
**Variables**	*OR*	*SE*
Age (ref. 65-74 years old)		
75+	1.14***	(0.04)
Health status (ref. good health)		
Poor health	1.11**	(0.08)
Gender (ref. male)		
Female	1.12**	(0.05)
Financial difficulties (ref. no)		
Yes	0.90*	(0.04)
Number of children (ref. one child)		
Two or more children	1.01	(0.04)
Change in health (ref. maintains health status)		
Experienced decline in health	1.69***	(0.07)
Depressed before (ref. no)		
Yes	1.01	(0.09)
Partner in household (ref. yes)		
No	0.90*	(0.04)
Educational level (ref. ISCED 0-2)		
ISCED 3-4	0.92	(0.02)
ISCED 5-6	0.88*	(0.04)
Intergenerational support (ref. No change or no support)		
Less often	0.92	(0.06)
More often	1.13*	(0.06)
European region (ref. Northern)		
Western	1.07	(0.08)
Southern	1.08	(0.09)
Eastern	1.01	(0.09)
Baltic states	1.28**	(0.11)
Observations	9,686	

**** p<0.001, ** p<0.05, * p<0.01*

Supplementary Table 4. Pooled OLS regressions (clustered SE) with country-fixed effects

	<u>**Pooled OLS 1**</u>	<u>**Pooled OLS 2**</u>	<u>**Pooled OLS 3**</u>	<u>**Pooled OLS 4**</u>	<u>**Pooled OLS 5**</u>
Age 75+ (ref. 65-74 years old)	0.99	0.99	0.99	0.99	0.98
	(0.03)	(0.03)	(0.03)	(0.03)	(0.03)
IADL limitations (ref. no IADL limitations)	1.01	1.01	1.01	1.01	1.00
	(0.04)	(0.04)	(0.04)	(0.04)	(0.04)
ADL limitations (ref. no ADL limitations)	1.05	1.05	1.05	1.05	1.05
	(0.05)	(0.05)	(0.05)	(0.05)	(0.05)
Chronic diseases (ref. no chronic diseases)	1.20***	1.20***	1.20***	1.20***	1.20***
	(0.04)	(0.04)	(0.04)	(0.04)	(0.04)
Female (ref. male)	1.59***	1.59***	1.60***	1.60***	1.60***
	(0.06)	(0.06)	(0.06)	(0.06)	(0.06)
Financial difficulties (ref. no)	1.29***	1.29***	1.29***	1.29***	1.29***
	(0.05)	(0.05)	(0.05)	(0.05)	(0.05)
More than one child (ref. one child)	0.92**	0.92**	0.92**	0.93**	0.92**
	(0.04)	(0.04)	(0.04)	(0.04)	(0.04)
Decline in health (ref. maintains health status)	3.92***	3.93***	3.92***	3.91***	3.91***
	(0.14)	(0.14)	(0.14)	(0.14)	(0.14)
Depressed before first outbreak (ref. no)	2.10***	2.10***	2.10***	2.10***	2.10***
	(0.07)	(0.07)	(0.07)	(0.07)	(0.07)
Without a partner in household (ref. with a partner in household)	1.10***	1.10***	1.10***	1.09**	1.09**
	(0.04)	(0.04)	(0.04)	(0.04)	(0.04)
Educational level ISCED 3-4 (ref. ISCED 0-2)	0.99	0.99	0.99	0.99	0.99
	(0.04)	(0.04)	(0.04)	(0.04)	(0.04)
Educational level ISCED 5-6 (ref. ISCED 0-2)	1.13**	1.13**	1.13**	1.12**	1.12**
	(0.05)	(0.05)	(0.05)	(0.05)	(0.05)
Germany (ref. Austria)	1.24**	1.16	1.16	1.15	1.15
	(0.13)	(0.12)	(0.12)	(0.12)	(0.12)
Sweden	1.12	1.13	1.14	1.10	1.11
	(0.13)	(0.13)	(0.14)	(0.13)	(0.13)
Netherlands	1.40**	1.30*	1.30*	1.29	1.29
	(0.21)	(0.20)	(0.20)	(0.20)	(0.20)
Spain	2.02***	1.85***	1.85***	1.85***	1.85***
	(0.22)	(0.21)	(0.21)	(0.21)	(0.21)
Italy	2.34***	2.02***	2.01***	2.02***	2.01***
	(0.24)	(0.23)	(0.23)	(0.23)	(0.23)
France	1.69***	1.50***	1.51***	1.49***	1.49***
	(0.17)	(0.16)	(0.16)	(0.16)	(0.16)
Denmark	0.84	0.83	0.83	0.82	0.82
	(0.11)	(0.11)	(0.11)	(0.10)	(0.10)
Greece	1.50***	1.47***	1.47***	1.45***	1.44***
	(0.15)	(0.16)	(0.16)	(0.16)	(0.15)
Switzerland	1.27**	1.29**	1.30**	1.28**	1.29**
	(0.14)	(0.14)	(0.14)	(0.14)	(0.14)
Belgium	1.72***	1.58***	1.59***	1.58***	1.57***
	(0.18)	(0.17)	(0.17)	(0.17)	(0.17)
Czech Republic	0.88	0.89	0.89	0.88	0.88
	(0.09)	(0.09)	(0.09)	(0.09)	(0.09)
Poland	1.35***	1.31**	1.31**	1.30**	1.30**

	(0.15)	(0.14)	(0.15)	(0.14)	(0.14)
Luxembourg	2.07***	2.15***	2.21***	2.15***	2.19***
	(0.27)	(0.28)	(0.29)	(0.28)	(0.29)
Hungary	0.88	0.84	0.84	0.85	0.84
	(0.14)	(0.14)	(0.14)	(0.14)	(0.14)
Slovenia	0.77**	0.80**	0.80**	0.79**	0.79**
	(0.08)	(0.09)	(0.09)	(0.09)	(0.09)
Estonia	1.28***	1.40***	1.40***	1.40***	1.39***
	(0.12)	(0.14)	(0.14)	(0.14)	(0.14)
Croatia	1.10	1.10	1.10	1.10	1.09
	(0.13)	(0.13)	(0.13)	(0.13)	(0.13)
Lithuania	1.24*	1.31**	1.31**	1.29**	1.29**
	(0.14)	(0.15)	(0.15)	(0.15)	(0.15)
Bulgaria	1.57***	1.68***	1.68***	1.66***	1.66***
	(0.20)	(0.22)	(0.22)	(0.22)	(0.22)
Cyprus	0.99	1.02	1.02	1.00	1.00
	(0.17)	(0.18)	(0.19)	(0.18)	(0.18)
Finland	0.94	1.01	1.02	1.01	1.01
	(0.13)	(0.14)	(0.14)	(0.14)	(0.14)
Latvia	0.79	0.84	0.84	0.82	0.82
	(0.12)	(0.13)	(0.13)	(0.12)	(0.12)
Malta	1.77***	1.74***	1.75***	1.77***	1.76***
	(0.24)	(0.24)	(0.24)	(0.25)	(0.25)
Romania	1.08	1.04	1.05	1.04	1.03
	(0.13)	(0.13)	(0.13)	(0.13)	(0.13)
Slovakia	0.94	0.90	0.91	0.90	0.90
	(0.15)	(0.14)	(0.14)	(0.14)	(0.14)
Time: Second outbreak (ref. first outbreak)	0.69***	0.76***	0.76***	0.75***	0.75***
	(0.02)	(0.03)	(0.03)	(0.03)	(0.03)
Intergenerational support: Less often (ref. no change or support)	1.12	1.11	0.28**	1.26***	0.34*
	(0.08)	(0.08)	(0.16)	(0.11)	(0.19)
Intergenerational support: More often (ref. no change or support)	1.70***	1.70***	1.72*	1.54***	1.17
	(0.06)	(0.06)	(0.51)	(0.07)	(0.37)
Stringency index at time of interview		1.01***	1.01**	1.01**	1.01*
		(0.00)	(0.00)	(0.00)	(0.00)
Stringency index * intergenerational support (ref. no change or support)					
Less often			1.02**		1.02**
			(0.01)		(0.01)
More often			1.00		1.00
			(0.00)		(0.00)
Excess mortality at time of interview		0.99**	0.99**	0.99***	0.99***
		(0.00)	(0.00)	(0.00)	(0.00)
Excess mortality * intergenerational support (ref. no change or support)					
Less often				0.97**	0.98*
				(0.01)	(0.01)
More often				1.02***	1.02***
				(0.01)	(0.01)

Constant	0.05***	0.03***	0.03***	0.03***	0.04***
	(0.01)	(0.01)	(0.01)	(0.01)	(0.01)
Observations	42,567	42,567	42,567	42,567	42,567

*Robust SE in parentheses; *** p<0.01, ** p<0.05, * p<0.1*

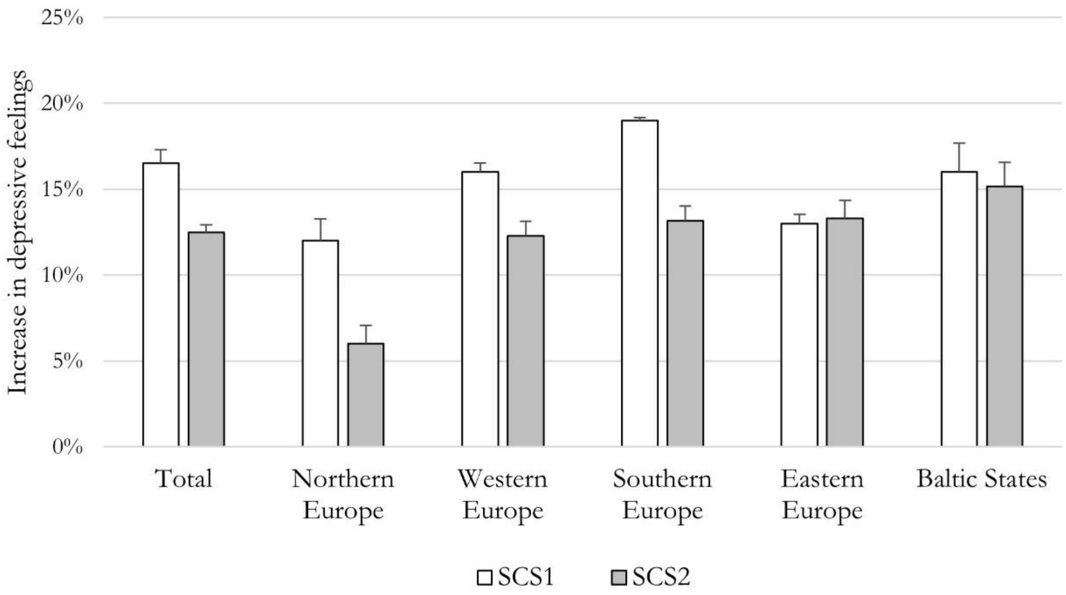

Supplementary Figure 4. Increased feelings of depression during SHARE Corona Survey 1 (SCS1) and SHARE Corona Survey 2 (SCS2) by region.

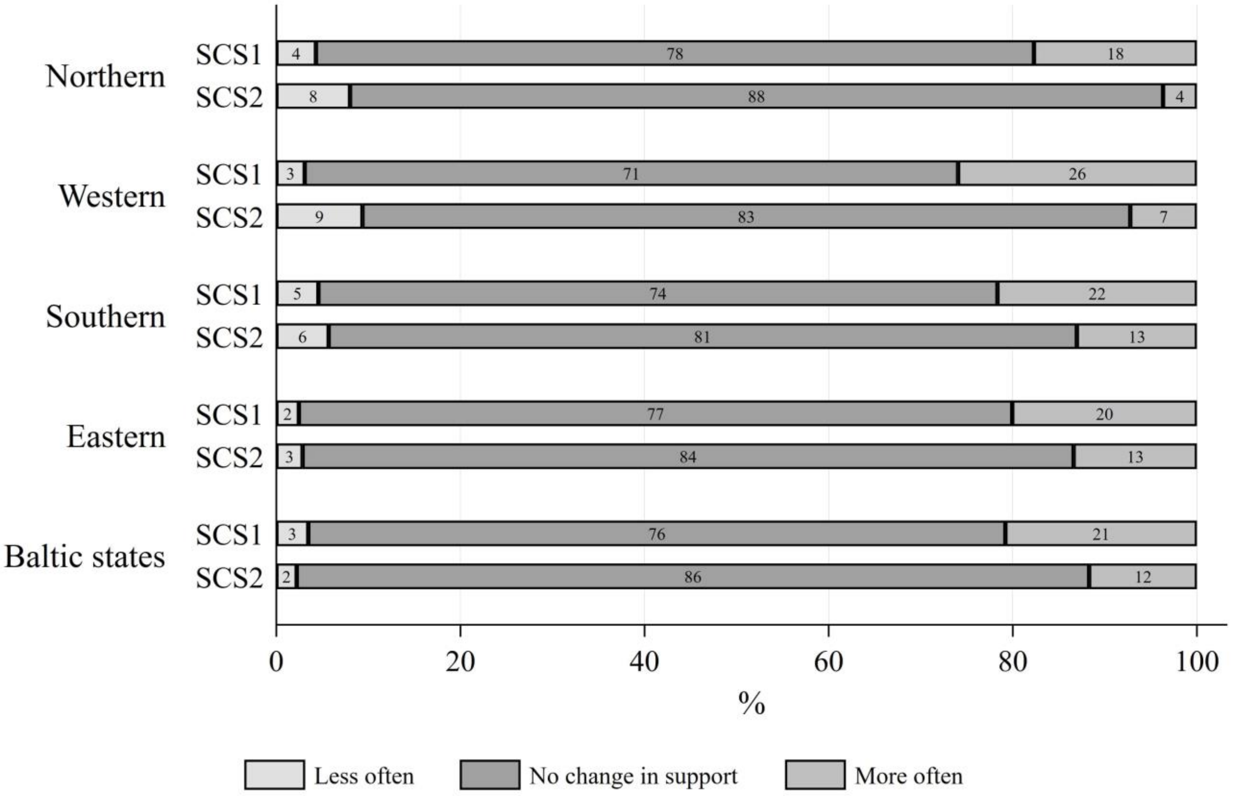

Supplementary Figure 5. Changes in receiving intergenerational support during SHARE Corona Survey 1 (SCS1) and SHARE Corona Survey (SCS2) by region.
